# Defining malaria risks among forest workers in Aceh, Indonesia: a formative assessment

**DOI:** 10.1186/s12936-020-03511-2

**Published:** 2020-11-30

**Authors:** Lenny L. Ekawati, Kelly C. Johnson, Jerry O. Jacobson, Carmen A. Cueto, Iska Zarlinda, Iqbal R. F. Elyazar, Abdul Fatah, Maria E. Sumiwi, Rintis Noviyanti, Chris Cotter, Jennifer L. Smith, Farah N. Coutrier, Adam Bennett

**Affiliations:** 1 Eijkman-Oxford Clinical Research Unit, Eijkman Institute for Molecular Biology, Jalan Diponegoro No. 69, Jakarta, 10430 Indonesia; 2 University of Berkeley, Berkeley, CA 94720 USA; 3 Malaria Elimination Initiative, Global Health Group, University of California San Fransisco (UCSF), San Fransisco, CA 94158 USA; 4 Malaria Pathogenesis Unit, Eijkman Institute for Molecular Biology, Jalan Diponegoro No. 69, Jakarta, 10430 Indonesia; 5 Aceh Provincial Health Office, Jalan Teungku Syech Mudawali No 6, Kota Banda Aceh, Aceh 23116 Indonesia; 6 United Nation Children’s Fund Indonesia, Jalan Jendral Sudirman Kavling 31, Jakarta, 12920 Indonesia

**Keywords:** Aceh, Malaria, High risk population, Socio-behavioural surveillance

## Abstract

**Background:**

Following a dramatic decline of malaria cases in Aceh province, geographically-based reactive case detection (RACD) was recently evaluated as a tool to improve surveillance with the goal of malaria elimination. While RACD detected few cases in households surrounding index cases, engaging in forest work was identified as a risk factor for malaria and infections from *Plasmodium knowlesi*—a non-human primate malaria parasite—were more common than expected. This qualitative formative assessment was conducted to improve understanding of malaria risk from forest work and identify strategies for targeted surveillance among forest workers, including adapting reactive case detection.

**Methods:**

Between June and August, 2016, five focus groups and 18 in-depth interviews with forest workers and key informants were conducted in each of four subdistricts in Aceh Besar and Aceh Jaya districts. Themes included: types of forest activities, mobility of workers, interactions with non-human primates, malaria prevention and treatment-seeking behaviours, and willingness to participate in malaria surveys at forest work sites and using peer-referral.

**Results:**

Reported forest activities included mining, logging, and agriculture in the deep forest and along the forest fringe. Forest workers, particularly miners and loggers, described often spending weeks to months at work sites in makeshift housing, rarely utilizing mosquito prevention and, upon fever, self-medicating and seeking care from traditional healers or pharmacies rather than health facilities. Non-human primates are frequently observed near work sites, and most forest work locations are within a day’s journey of health clinics. Employers and workers expressed interest in undertaking malaria testing and in participating in survey recruitment by peer-referral and at work sites.

**Conclusions:**

Diverse groups of forest workers in Aceh are potentially exposed to malaria through forest work. Passive surveillance and household-based screening may under-estimate malaria burden due to extended stays in the forest and health-seeking behaviours. Adapting active surveillance to specifically target forest workers through work-site screening and/or peer-referral appears promising for addressing currently undetected infections.

## Background

Since national and provincial malaria elimination plans were launched in 2009, the annual parasite incidence (API) in Aceh province, situated at the northwestern corner of Indonesia’s vast archipelago, has declined greatly, from 0.9 to 0.1 cases per 1000 population between 2010 and 2015 [1, 2]. In the spatially progressive national malaria elimination strategy, Aceh, with a population of 5.2 million, is envisioned as an early milestone, both due to its geographic position and for its early successes in strengthening malaria control following the 2004 Indian Ocean Earthquake and resulting tsunamis. As of 2017, around 78% of Aceh’s 23 districts were certified as malaria free. However, locally acquired malaria cases have continued to occur past Aceh’s initial 2015 elimination target. In Sabang district, a group of islands in Aceh north of the mainland that was previously one of the most malarious areas in Indonesia, there were no cases of *Plasmodium falciparum* and *Plasmodium vivax* after 2011. However, several locally acquired cases of *Plasmodium knowlesi*, a non-human primate parasite that is more difficult to diagnose by microscopy, were detected in 2014 [3]. Outside of Sabang, *P. falciparum*, *P. vivax*, *P*. *knowlesi* as well as mixed infections continued to occur, with 37 cases in 2016.

The large declines in incidence to date have been achieved by strengthening the capacity of health facilities for diagnostics and treatment with artemisinin-based combination therapy, community-based distribution of long-lasting insecticide-treated nets (LLINs), systematic mapping of cases, and interventions targeting areas of greater endemicity, including indoor residual spraying (IRS), improved vector management, and active case detection (ACD) by testing of symptomatic individuals at households and schools [2–4]. As malaria caseloads declined, reactive case detection (RACD) was introduced to detect remaining infections in 2010 in Sabang and in 2014 in Aceh Besar, a low-endemic district with 78 cases and an API of 0.21 in 2014 [3, 5]. RACD aimed to screen all family members and neighbors within 500 m of the households of index cases [6]. While RACD in Aceh Besar yielded just six secondary cases (0.4%) among 1495 individuals screened over a 19-month period [3], survey data collected from those screened suggest that transmission may be occurring in the forest rather than in communities: risk factors for malaria included being male, engaging in forest-related work, and staying overnight in or near the forest [3]. Moreover, *P. knowlesi* was the most common parasite identified. If transmission occurs primarily in the forest, current strategies of passive surveillance and ACD in communities could fail to detect infections if forest workers do not seek care at public health facilities and are frequently away from their homes [3]. Indeed, evidence across the region shows that household RACD may detect few cases in a setting of forest transmission and is unlikely to lead to elimination of parasite reservoir [7].

Across the Asia–Pacific region, forest activities such as agriculture, logging and gold mining are considered high-risk occupations for malaria exposure [8]. Moreover, forest workers in this region tend to exhibit patterns of mobility between endemic and non-endemic areas that may contribute to sustaining transmission and re-introducing malaria where previously eliminated [9–12]. Although approximately half of Aceh’s land area is occupied by forest [13], there has been little systematic examination of forest activities to determine how to include forest-going populations workers as part of the malaria elimination strategy.

This qualitative study aimed to characterize forest activities, identify subgroups of forest workers that are likely to be at risk and their mobility, and identify strategies for improving surveillance in these subgroups, including targeted active surveillance approaches. Regarding surveillance, the study aimed to determine whether they are likely to seek care at the public health facilities that comprise the passive surveillance system, and determine how to access high-risk subgroups for screening and prevention.

## Methods

### Study setting

**Fig. 1:**
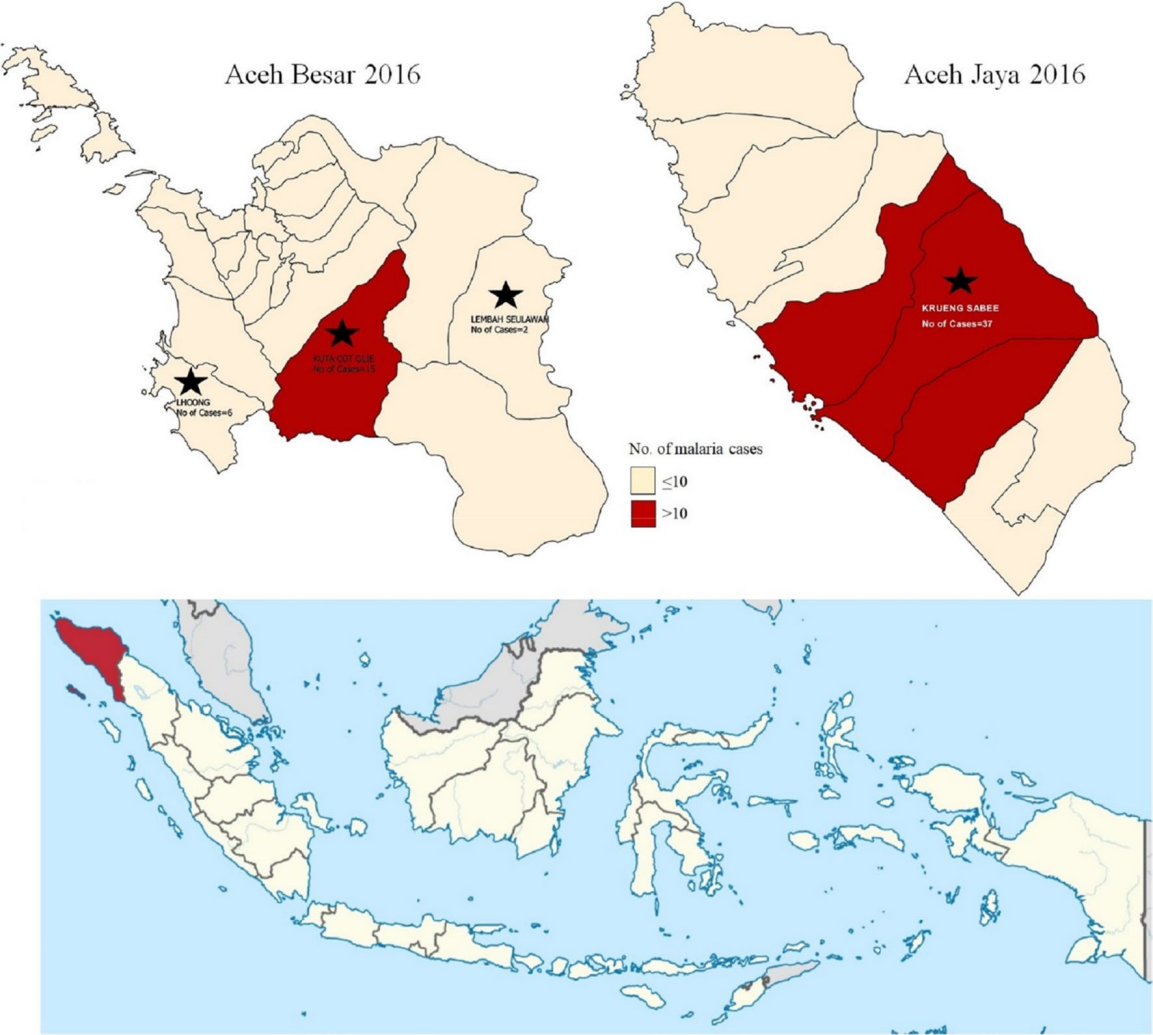
Four study sites in Aceh province, Indonesia

The study was conducted in four subdistricts in Aceh with high levels of forest cover and suspected malaria exposure through forest work: Lhoong, Saree and Kuta Cot Glie subdistricts in Aceh Besar and Krueng Sabee subdistrict in Aceh Jaya district (Fig. 1). Aceh Besar has an average temperature range from 26 °C to 28 °C and an area of 2903 km^2^ with 23 sub-districts and 604 villages and a 2015 population of 392,584 inhabitants. Malaria transmission peaks from January to July, while August to December tend to see few cases. Public health services include 28 primary health centres (PHC) and 69 sub-PHCs. Lhoong and Kuta Cot Gile represent two of the five subdistricts in Aceh Besar where household RACD was previously piloted [2]. Most residents in these two subdistricts work in agricultural, manufacturing and service sectors [3]. Krueng Sabee of Aceh Jaya district comprises 588 km^2^ of land area with a population of 15,937 inhabitants living in 17 villages that is served by two PHCs and three sub-PHCs. Residents work mainly in agriculture, industry and trade [14].

### Study participants and recruitment

Recruitment and data collection took place between June and August of 2016. Study key informants (KIs) consisted of nine groups: index cases and their co-workers, community health worker, community member who lived in the forest fringe and non-forest fringe, employer of forest workers or *toke*, health staff who work in health facility, and community leader. This study implemented a combination of sampling methods: consecutive sampling of historical passively detected index cases, snowball sampling of referrals and convenience sampling of community leaders/individuals in positions with specific knowledge of forest-going populations and malaria health services.

All individuals who had been diagnosed with malaria at a PHC between 2014 and 2015, resided in one of the four sub-districts during the prior transmission season, and were aged 18 years or older were identified as “index cases” and invited to participate in either Focus Group (FG) or In-Depth Interview (IDI). Consenting index cases for FG were requested to provide contact information for 2–3 other individuals, aged > 18 years, with whom they had visited the forest in the past two years. This type of KI was defined as “co-workers” and only participated in FG with index cases.

For focus group, the study coordinator contacted community members who were knowledgeable about forest work, and community health workers, such as village health cadres who were familiar with forest-working activities and malaria diagnosis. Community leader who widely perceived to represent the community and government authorities or non-governmental organizations (NGO), medical doctors and nurses from primary health centres were identified and invited to participate in IDI. Index cases and community leaders were asked to provide contact information for forest work employers or *toke* to be offered for their participation in the IDI.

### Data collection and analysis

In each subdistrict, five FG of 6–10 KIs each were carried out with index cases and their referred co-workers (N = 2), community health workers (N = 1 FG), residents of forest fringe villages (N = 1), and non-forest fringe villages (N = 1). A total of 20 FGs were conducted with 173 KIs across the four study sites. In parallel, 72 IDIs were held in each subdistrict with index cases (N = 5 IDIs), *tokes* (N = 3), community health workers and NGO staff (N = 2 to 5), health facility staff (N = 2), and community leaders and government authorities (N = 4 to 5).

Some authors in this paper linked to the previous malaria work in Aceh province [3]. In a further collaboration with qualitative researchers, the questions in the formative assessment were composed (Additional file 1). Beside inquiring about phenomena, understanding and perception about malaria risks, the questions also shaped the questions into more specific field study [15]. Most qualitative questions focused on the local and “the thick description” which describes human interactions in that context [16].

The IDIs and FGs were performed by trained local interviewers in the Acehnese language. All interviewers underwent a 1-week training that focused on qualitative techniques for interviewing and facilitating FGs; emphasis was placed non-judgmental elicitation of responses and probing to improve exploration of the research questions. In that session, the researchers employed pilot or member-checking procedures to seek objective opinion as to how questions could be made easier to understand, avoid bias or leading questions and/or avoid any potential ambiguity. Training included mock interviews to gain experience with and refine the data collection guides and study procedures.

IDIs and FGs were used to elicit complementary information by ensuring representation of both individual experiences and group processes exploring themes. The semi-structured interview guides tailored to each respondent group, which explored work and mobility patterns in the forest as well as behavioural exposures related to malaria. In addition, interviewers detailed two potential methods for conducting screening and/or behavioural surveys of forest workers [17], including peer-referral (PR) and venue-based (VB) recruitment; participants were then asked to discuss the feasibility of each method.

IDIs and FGs lasted on average 48 and 63 min, respectively. Audio recordings of the IDIs and FGs were transcribed by research assistants verbatim in Acehnese. The first author translated all transcripts to Bahasa Indonesia and developed an English language summary of each transcript, which highlighted key themes related to each research aim. The first and second authors reviewed all summaries and selected transcripts which mostly answered the key themes for full translation into English by an independent translator.

A draft codebook was developed that included both a priori codes from the interview guides and themes that emerged from initial reviews of the summaries and transcripts. The first and second authors used the draft codebook to each independently code transcripts using the qualitative analysis software Dedoose ver. 8 (California, U.S.A). Following the method employed by MacQueen et al. the coding was compared side by side and discussed for the differences, before the codebook was further refined and finalized [18].

Thematic analyses were conducted on the transcriptions of participants’ responses to interview questions and determine themes [19]. Themes were extracted from the data following a grounded theory approach [20], in which the qualitative data were repeatedly explored, interpreted, and categorized, and consistency of the findings was sought by comparing information from different sources.

In order to identify themes, the first and second author searched the data for the following occurrences and patterns: repetition (reoccurring topics or concepts), metaphors and analogies used by participants, indigenous typologies (“local” terms used by the participants), and paradigm cases (particularly vibrant examples that stand out from the other text, and which embody the meaning of participants’ practices) [21]. The first and second authors subsequently collated data excerpts based on these emergent categories and assessed for discrete themes and sub-themes within each category. Themes were discussed by the first and second author and increasingly refined and collapsed into higher-level themes.

Maps of worksites in each subdistrict were developed based on the content of the FGs and IDIs, denoting the location, distance and travel time from nearest village, and type of work activity at each site.

## Results

Treatment-seeking behaviour is defined as sequence of actions that taken by individual in responding to their illness. Many people, including forest workers differ in their willingness to seek health assistance. Some are immediately ready for early diagnosis and expect proper treatment. Others probably only when in a great pain or in advance state of their illness. Following qualitative analysis, this section presents the characteristics of the informants, occupational groups among forest workers, and themes of analysis, such as malaria beliefs and risk behaviour, treatment-seeking behaviours, methods to access forest workers and future participation.

### Description of participants

The study included 231 participants, with approximately equal numbers across the four subdistricts (Table 1). Median age of male forest workers was 30 years (range 18 to 56), whilst median age of other KIs was 36 years (range 19 to 73) with a male to female ratio of 1.8 to 1. Forty-one individuals (34 males and 7 females) contacted declined to participate due to work activities in the forest (17), immediate family matters (11), uncontactable (8), and unwell (5) on the assigned day of group discussions.

**Table 1:** Characteristics of study participants

Characteristics	Aceh Jaya	Aceh Besar	Total		
	Krueng Sabee	Kuta Cot Glie	Lhoong	Saree	
Study participants	57	56	59	59	231
Median of age (range)	35 (19–73)	33 (19–55)	33 (18–59)	36 (22–53)	34 (18—73)
Male: female ratio	44: 13	45: 11	47: 12	44: 15	180: 51
Patients and co-workers					
Farmer	5	10	7	19	41
Logger	–	9	14	–	23
Miner	15	1	–	–	16
Driver	–	2	1	–	3
Chips seller	–	–	–	1	1
Forest ranger	–	–	–	1	1
Police	–	-	–	1	1
Community health workers					
Health cadre	11	8	10	12	41
Owner local pharmacy	–	2	1	1	4
Midwife (private practice)	–	1	–	–	1
Community leader					
Village leader	2	2	4	2	10
Respected leader	2	2	1	2	7
Religious leader	1	–	–	–	1
NGO	1	–	–	–	1
Health facility					
General practitioner	1	1	–	2	4
PHC staff	1	1	1	–	3
Midwife	–	–	1	–	1
Forest community					
Farmer	3	7	5	7	22
Miner	3	–	–	–	3
Construction worker	–	–	1	–	1
Small enterprise	–	–	1	–	1
Non-forest community					
Farmer	2	2	3	8	15
Small enterprise	4	2	2	–	8
Fisherman	–	–	4	–	4
Student	1	2	–	–	3
Government employee	1	1	–	–	2
Driver	1	–	–	–	1
Employer or *toke*	3	3	3	3	12

### Occupational groups

**Fig. 2:**
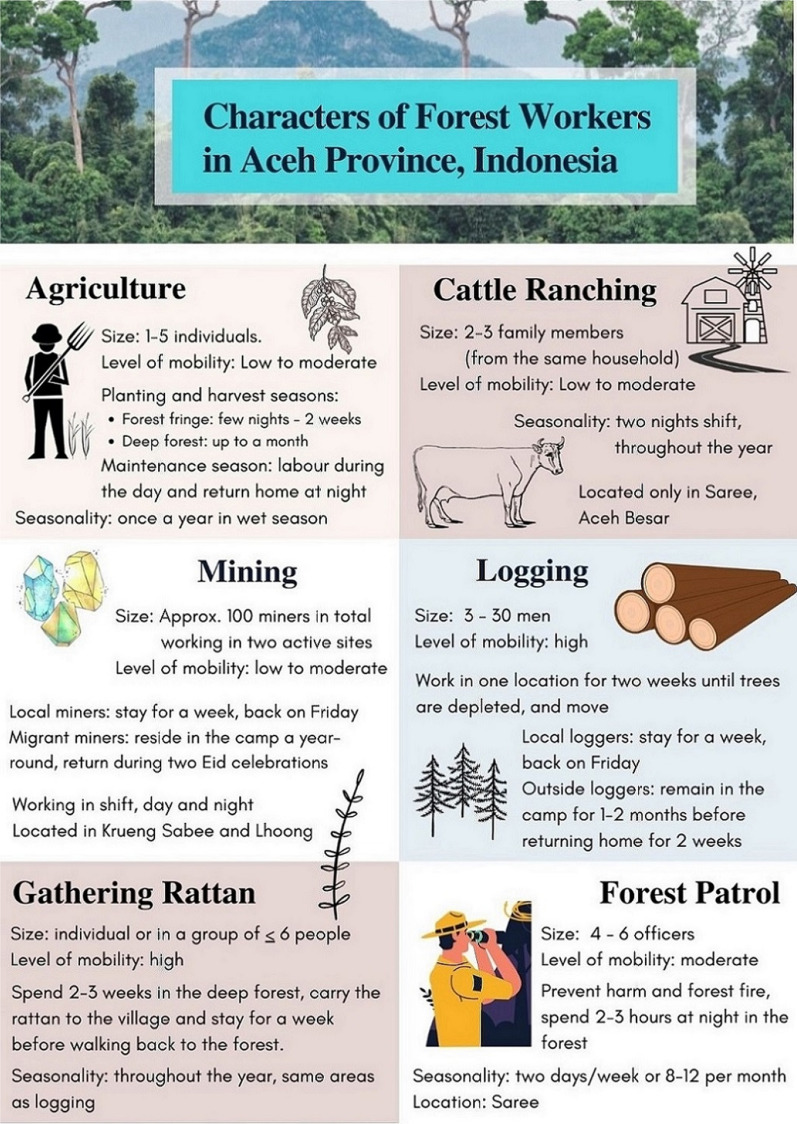
Primary forest activities in four study sites in Aceh province, Indonesia

KIs described six main activities that occur in forested areas: agriculture; cattle ranching; logging; mining; gathering rattan; and forest patrol (Fig. 2). Agriculture was reported in all sub-districts and was small scale, with work groups of 1 to 5 individuals (4 to 5 during planting and harvesting seasons) typically from the same village. Crops cultivated in forest fringe areas included corn, rice, chili, candlenut, cassava and areca nuts, clove, nutmeg, sweet potato, yam, peanut, and eggplant, while durian, cacao, banana, and coffee are generally grown deeper in the forest. Agricultural forest workers typically labour during the day and return home to their villages at night, except during the work-intensive planting and harvest seasons, when overnight stays at plantations become more common. Farmers who are unable to return home each evening reported spending from a few nights to 2 weeks at forest fringe plantations and up to 1 month at plantations located deeper in the forest, in part to work and in part to guard their crops from animals. During these periods, farmers sleep in simple huts.

**Fig. 3:**
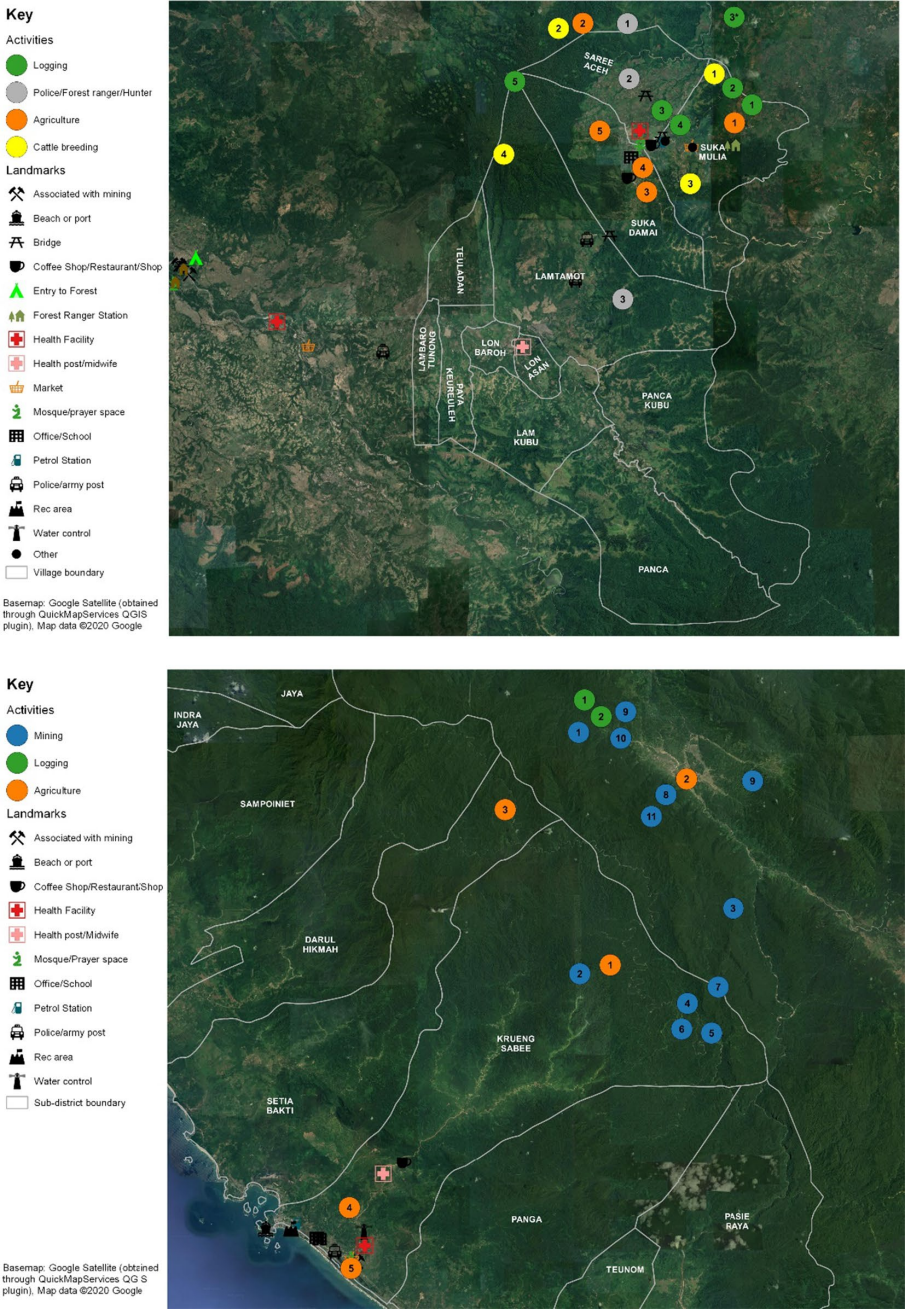
Activity maps reported by the forest workers in Saree and Krueng Sabee. Illustrates activities in agriculture (orange), logging (green), mining (blue), cattle breeding (yellow) and police/forest ranger/hunter (grey). Base map source: Google Satellite, obtained through Quick Map Services QGIS plugin. Map data @2020 Google. Access date: January 9, 2020

Figure 3 illustrates that cattle ranches in forested areas were only reported in Saree; they are located in the forest fringe and collectively owned by villages. Residents take turns spending the night (in 2-night shifts) at the ranches to guard cattle, generally in groups of 2 to 3 family members from the same household.

Logging was reported by KIs in Kuta Cot Glie and Saree, at forest work sites that typically comprise from 3 to 30 men and shift location frequently; loggers were described as staying at a given site for about 2 weeks until trees are depleted before moving elsewhere. Because forest work camps are generally located from 2 to 6 h by vehicle from the nearest village, followed by 30 min on foot to the logging work site, loggers who reside within Aceh typically sleep at the logging camp for a week at a time, returning home on Fridays to pray and purchase the logistics and food for the group. Loggers who reside outside of Aceh (typically North Sumatra or West Java) often remain at the logging camp for 1 to 2 months before returning home for about 2 weeks.

Gathering rattan (harvested from wild climbing palms belongings to sub-family Calamoideae are used for baskets and furniture) was also described as a frequent activity in Kuta Cot Glie and Krueng Sabee; this activity occurs throughout the year in the same areas as logging. Workers frequently work alone or in a group of up to six people, and the gatherers spend 2–3 weeks in the deep forest, then carry the rattan to the village and typically stay a week before returning to the forest.

Participants described two mines in the forests of Krueng Sabee and Lhoong, which employed approximately 100 labourers in total. The Krueng Sabee mine is located two hours by vehicle from the nearest village. Miners included both local (from within the district), and migrant workers (travelling from elsewhere in Aceh Province, West Sumatra and several provinces in Java). The local miners usually stay at the mining camps during the week and return home on Fridays, whereas migrant miners generally reside at the camps year-round, returning home only during the two annual Eid celebrations (Eid al-Fitr at the end of Ramadan and Eid al-Adha approximately 2 months later). Miners reported sleeping near their mining site.

The Lhoong mine is located 20 min from the nearest village on the forest fringe and is owned by a foreign company that has ceased operations; however, about 20 residents of local communities often enter and mine there illegally during the evenings, returning home each day to process material at a plant located in the village.

One KI was a police officer who trained newly recruited cadets in the police academy in Saree. Over a period of 2 to 4 months, he conducted annual intensive training for around 300 cadets which required him to march on foot and spend nights in either military tents or on the ground under the open sky along with 50 trainees. Apart from regular training activities, police officers perform routine and 24-h patrol shifts at the base.

The main tasks of forest rangers include patrolling in forested areas to prevent harm and forest fire, and raising awareness to prevent illegal logging. In a group of 4–6 officers, they routinely patrol their remote posts –located around six kilometres from the outermost village– for 2–3 h at night. Forest rangers in Saree generally work two days per week or 8–12 days per month from 9 a.m to 5 p.m.

### Malaria beliefs and risk behaviours

Across study sites, most participants perceived forest workers to be at high risk for malaria. Some participants believed that malaria risk was limited to people who work and stay overnight in the forest or mountains, and stated that individuals living in villages were rarely infected. Many participants reported that spending time in cold places and sleeping out in the open air or in a simple hut without using mosquito net were the main reasons people contract malaria. The majority of participants perceived malaria patients to be predominantly men, with a much smaller percentage of children:

*As far as I know there are several villagers here who got malaria according to blood test results. Most of them are farmers who work in the fields, most are male, but some are children… but most are male because they often have to sleep out in the open air in the field, they don’t use mosquito net (Participant SR004, male, 39, community leader)*.

At all study sites, forest workers described the presence of monkeys, which they reported witnessing during daytime travel within the forest. Most reported seeing long-tailed macaques (*Macaca fascicularis,* Local name: *Bukreh*), southern pig-tailed monkeys (*Macaca nemestrina,* Local name: *Siben or Beruk*) and Sumatran surili (*Presbytis melalophos,* Local name: *Buduk or Budeng*). Several participants reported that monkeys often come to the logging sites or to forest plantations at night to eat crops during harvest season. Most participants declared there was no interaction between the workers and the monkeys, while a small number of workers reported feeding them fruit, capturing them for sale, bringing them home as pets or chasing them away.

Almost all participants described malaria symptoms (N = 279) as one or more of the following: headache (39), chills (33), fever (31), muscle and joint pain (30), feeling hot and cold or *sijuk suum* (29), weak (21), vomiting (11), nausea (8), and dizziness (7).

Most participants reported low rates of prevention practices among forest workers, while several reported the use of bed nets, mosquito coils, repellent and medication to prevent malaria symptoms. The most common behavioural risks perceived were not using mosquito nets or repellent at night, working and sleeping outdoors at night, and doing no more to ward off mosquitoes when sleeping than lighting a fire.

*I don’t think we would feel anything if a mosquito bit us… If we remember, we apply [repellent]. Sometimes if we get too tired after marching from morning until afternoon, we fall asleep fast. (Participant SR016, male, 30, police officer).*

Many participants believed that eating certain foods protects against malaria, such as papaya leaves, bitter melon [local name: *paria or pare*], mahogany seeds, cats’ whiskers, and lanzone root [local name: *langsat*]. Others mentioned traditional herbs or *jamu*, made from leaves of king of bitter [local name: *bratawali*] and long jack leaves [local name: *pasak bumi* or *tongkat Ali*]. Wearing charmed stones was seen as protecting against mosquito bites.

Many participants said they did not regularly use bed nets because they made breathing difficult or were hot and uncomfortable. Some believed that chemicals used to treat the nets resulted in odours that irritate the eyes and skin. A number of participants who were willing to utilize the mosquito nets in their working areas lamented that they were expensive, often out of stock, or were not distributed specifically to forest workers. Some forest workers viewed prevention as unnecessary because they can buy medication whenever they feel ill.

### Treatment-seeking behaviours

Forest workers described a variety of strategies for fighting off malaria symptoms when they experienced them in order to continue working. Many reported that they tended to ignore symptoms or self-treat with medications they brought from local pharmacies as a precaution when travelling into the forest, including analgesics, antipyretics, food supplements and anti-malarial drugs.

When malaria symptoms persisted after self-treatment, the workers described visiting a pharmacy as soon as they arrived in the village and purchasing the commercial brand of chloroquine without being tested for malaria.

*Bayer. Sometimes we bought Paramex [an analgesic], but when we arrived to the mountain we didn’t need that because we were sick from something else. No, they [people at the pharmacy] don’t check for it [malaria]. We ask them to give us some medicines as a precaution (Participant KG101, FG, patients and their co-workers).*

While some KIs reported that malaria patients seek care at PHC’s, participants more often described presenting to the nearest private clinic, often run by a nurse or midwife. Although private clinics rarely have laboratory facilities available, they were seen as more convenient because they were open in the evening after work, conducted examinations quickly and were run by well-known people. Private clinics were also perceived to be effective at treating patients.

*They [the private clinic] will check the patient’s health condition and then give an injection. The sick usually gets better in one night. (Participant KS008, male, 40, toke)*.

Other participants reported that forest workers often consulted traditional healers before going to the PHC, particularly when they or their family believed that malaria was by an evil spell or by a bad spirit encountered at a work site. Some participants saw traditional healers as both affordable and effected, and trusted in their treatments to effectively repel the disease, which included written prayers, figure or images with religious or mystical properties [local: *jampi-jampi* or *rajah*]. Two participants, a community member and *toke*, stated that some private clinic staff also refer patients to traditional healers.

*It’s what we believe… It’s our tradition, to first find out whether the sick person is possessed by an evil power or not. That’s why we bring the patient to a traditional healer, to get rid of the evil power… and the patient doesn’t have to pay much either (Participant KG105, FG, forest fringe community).*

One of the primary barriers for seeking medical treatment among forest workers was the high cost of transportation to health facilities from forest work sites or their homes. One KI stated,

*One of the reasons I didn’t go to primary health centre perhaps because it’s too far from my house. Not all people here have a car. So first aid is by taking traditional herbs (Participant LH101, FG, patients and their co-workers).*

In addition, some participants perceived PHC’s to have inconvenient operating hours as well as unhelpful, disrespectful or untrustworthy staff.

*When people go to PHC, sometimes staff are ignorant. They receive many complaints from the villagers. Perhaps people who go to PHC are lower middle class and PHC staff are disrespectful. There are two to three people there [at the PHC] who seem unmotivated to provide the services. We are not too happy. There a case… it happened 2 months ago… one of the doctors gave a wrong diagnosis (Participant SR004, male, 39, community leader).*

Furthermore, stock outs were cited as a barrier for seeking care at PHC’s: one KI (a police officer) reported stock outs of anti-malarial medications at a police academy clinic.

*We are out of [anti-malarial] medicines right now… I think I saw some quinine pills available there…I don’t know what that is, but we rarely use them (Participant SR016, male, 30, police officer).*

Among migrant workers, seeking treatment at PHC’s was reported to be more complicated because they were not registered in the local health insurance scheme. As a result, several participants reported that migrant workers tend to prefer private clinics in cases of emergency. One toke who employed migrant workers explained why he chose to take his employees to private clinics instead of to the PHCs:

*Sometimes we employ workers from outside this village, and I cannot take care of everything because the procedure is too long. They [staff at the PHC] ask for family card identification, this and that. It’s a long procedure. We need a quick medical treatment because we bring a sick person here (Participant KS018, male, 40, toke).*

A number of participants informed that forest workers avoided seeking treatment at PHCs due to a fear of needles and misconception about injected treatment for malaria. Others reported that they preferred to first seek help from traditional healers, particularly if their symptoms are mild.

*Before going to Puskesmas, we usually see a traditional healer [Local: dukun]. That’s because we don’t know what we have… Why we are sick. People say if we fell into a trance, we cannot be injected…. It’s being possessed by an evil spirit… touched by a ghost (Participant LH102, FG, patients and their co-workers).*

Several community leaders, *tokes* and forest workers viewed PHCs and hospitals as preferable when illness was severe or potentially fatal, or when symptoms worsened following initial treatment elsewhere, especially because services at the government health centres are free of charge. Once at a PHC, participants recounted receiving laboratory confirmation of malaria prior to receiving treatment.

Participants were generally unwilling to speak about illegal forest workers. However, some speculated that they do visit the PHC and sometimes have misconception about malaria treatment.

### Methods to access forest workers

Determination of whether VB and/or PR-based surveillance were likely to be feasible methods to access forest workers was based on themes that emerged from the data (Table 2). VB was considered a practical option for occupational groups that tend to stay overnight or work at well-defined work sites during mosquito biting hours that are geographically accessible, safe to visit and accessible, such as miners. PR was considered for groups whose members tend to interact with one another frequently and are likely to be willing and able to present to a surveillance officer located in a village or settlement within 2 to 3 weeks of referral.

**Table 2:** Possible surveillance approaches for particular forest occupational groups

Occupational Group	Aceh Jaya	Aceh Besar		
	Krueng Sabee	Kuta Cot Glie	Lhoong	Saree
Farmer	Peer-referral	Peer-referral	Peer-referral	Peer-referral
Rancher	–	–	–	Peer-referral
Miner	Venue-based	–	Venue-based	–
Logger	–	Venue-based and/or Peer-referral	Venue-based and/or Peer-referral	Peer-referral
Rattan gatherer	Peer-referral	Peer-referral	–	–
Forest patrol/police	–	–	–	Venue-based

Agricultural workers, rattan gatherers and rangers were seen as accessible by PR because they often hail from the same village and know one another, whereas efforts to recruit at work sites may confront several challenges: individuals tend to work alone or in small groups that do not work daily or during predictable schedules, and are not organized by *tokes*; those who return home in the evenings would generally be unavailable working during the day and travel to more remote work sites would be infeasible during the rainy malaria season.

*The area is difficult to reach and also very far. There’s a chance too that the people you’re going to see are not available that time. It is difficult also to communicate with them because there is lack of communication device… Besides that, it’s very difficult to reach the place (Participant SR102, FG, forest fringe community).*

Because cattle ranchers operate collectively in a well-organized group, PR or a screening event held in the community coordinated by *tokes*, village leaders or local midwives were seen as promising options, while participants did not view VB as an ideal plan for conducting a study.

*It’s not a suitable place to meet at work place, it’s better to meet here [in the village], so it can be explained to them…what malaria drug is, how we can prevent malaria. We can arrange the meeting in Meunasah [local: small mosque] … or…in the village hall (Participant SR005, male, 53, cattle toke).*

In contrast, PR was not seen as possible for gold miners in Krueng Sabee, because local and migrant subgroups often do not interact with each other. However, VB was reported to be a feasible and acceptable option, as miners are likely to be accessible at the work site: they reside there during extended periods, and therefore could be accessed when off shift. In addition, the *tokes* interviewed at the mines expressed willingness to facilitate access for screening purposes.

Furthermore, mining camps are reachable within two hours from the district centre, which would facilitate the transportation of equipment and supplies required for a VB intervention. *Tokes* suggested the importance of gaining support of village leaders when planning VB screening. Of note, females are forbidden at the mining camps because they are thought to harbor bad luck, which would preclude female nurses and other staff from participating in VB surveillance.

A routine malaria examination every 3 months was viewed as beneficial for forest workers and their employers; *tokes* also strongly requested feedback on the malaria test results.

*When you come to check their health condition, the workers would know about their health condition…. If during the medical check there are symptoms of sickness, they will know it too. But we have never had our health condition checked, there’s no explanation about health issues before (Participant KS018, male, 40, toke).*

However, loggers in Lhoong and Kuta Cot Glie were seen by most participants as unreachable at their work sites, which tend to be remote and difficult to access. In addition, some participants reported that the presence of VB surveillance staff in the forest would be viewed as suspicious by loggers.

*"It’s not possible to ride a motorcycle passing that road. They would think negative things… [such as] why do these people come here, are they logs buyer? It would raise questions (Participant LH105, FG, forest community).*

Finally, participants reported that loggers would not want to be distracted from their work, and therefore would not be interested in participating in VB surveillance. The majority of participants also viewed PR as an infeasible strategy for reaching loggers. Participants reported that local and migrant loggers tend to not interact with each other, and most migrant workers do not stay in local communities upon leaving the work site. *Tokes* were supportive of PR, however, and offered to assist with surveillance by making workers available and inviting them to meet at a workshop facility in the local village or at the *toke’s* residence. Because *tokes* change frequently and one participant felt community leaders should also be involved in helping to plan for PR surveillance.

*The toke will contact the workers, because we don’t know the workers ourselves…they are not from here…all of them are outsiders (Participant KG105, FG, forest community).*

In Saree, where the majority of the forest is protected, KIs reported that most logging that occurs is illegal. Because of this, loggers and *tokes* in Saree would not be amenable to work site screening. KIs reported that illegal loggers would be open to participating via PR, but they would probably not admit to engaging in logging and would instead say they were doing other activities in the forest. For police cadets and forest rangers, however, venue-based screening is more likely feasible compared to the other.

### Future participation

Most informants expressed interest in participating in future malaria surveillance activities, provided they do not interfere their work. All occupational groups preferred cash incentives, with desired minimum amounts varying from USD 3.85 to 7.69, in addition to compensation for any transportation cost to travel to the study location.

## Discussion

This study is the first to describe and characterize forest workers and their malaria risk in Aceh province where they engage predominantly in logging, mining and agricultural activities. When these groups experience illness, they prefer self-treatment or seek traditional healers before presenting to the nearest health care facility as ailments worsen. Their treatment-seeking behaviours provide insights for the Indonesian malaria programme, as the country prepares for malaria elimination in 2030. Globally, as national malaria programs seek to address suspected high-risk groups with which they have little previous familiarity, rapid, formative assessment studies such as this one are an increasingly relevant first step to crafting effective elimination strategies [8, 15], as they have been in the HIV context [22–24].

Findings from qualitative work showed that lack of understanding about malaria risk may be influencing treatment-seeking behaviours. Moreover, in the context of forest work, language, beliefs about malaria, trust in authorities, and perceptions of local health care systems can facilitate or obstruct ones willingness to seek treatment [25]. Forest workers reported that they purchase available anti-malarial and other drugs over the counter at local pharmacies in order to self-treat malaria-like symptoms. Following symptom onset, they often delay seeking treatment when the illness is mild or immediately consume stock medications as self-treatment.

Misconceptions around malaria risks, prevention and injections for malaria treatment among individuals in the study suggests a greater need for health education, both at work sites and in villages [26]. Similar to previous findings [27, 28], these results suggest insecticide treated bed nets may have limited impact for forest workers because they are impractical to set up and uncomfortable to sleep under. As an alternative, studies in Vietnam and elsewhere have evaluated long lasting insecticidal hammocks (LLIH) [29].

For those who experience malaria-like symptoms, treatment of malaria should be taken after positive confirmation by either microscopy or rapid diagnostic test (RDT) [30]. In addition, health promotion, knowledge of prevention measures by forest workers are potentially inhibiting factors to the spread of malaria [31, 32].

This study suggests that more aggressive active surveillance is needed to track and treat both symptomatic and asymptomatic infections circulating in high-risk groups such as forest workers [28]. At worksites, *tokes* appear motivated to participate because they value keeping their workers healthy. In previous studies, *tokes* were also generally misinformed about malaria risk. Providing *tokes* and forest workers with prevention education could improve and motivate them to participate in future surveillance efforts.

This study also examined the feasibility and acceptability of potential surveillance approaches for addressing high-risk populations in Aceh, including PR and VB. Overall, PR was considered a feasible option for groups whose members tend to interact with one another frequently and are likely to be willing and able to present to a study office located in a village or settlement within 2 to 3 weeks of referral. VB was considered a practical option for occupational groups that tend to stay overnight or work at well-defined work sites during mosquito biting hours that are geographically accessible, safe to visit and accessible.

The study findings recommend PR for farmers, VB screening for miners and a combination of PR and VB strategies for loggers. Farmers, village leaders and local midwives expressed willingness to be contacted directly; accessing miners and loggers needs to be done in partnership with the *tokes* and potentially off-site. Engaging staff from PHCs such as medical doctors, nurses and midwives is critical to set up VB visits in order to gain trust, and easy access, and to ensure credibility.

Many migrant workers sought treatment in private clinics because they lacked local health insurance and these clinics offered more flexible admission times, especially when the workers arrived from the forest after office hours. Study findings support that the provincial and district health offices should register private clinics which often receive patients with malaria-like symptoms. Additionally, authorities should regularly ensure reliable RDT inventory for registered private clinics. When a patient present to a clinic with malaria-like illness and has a history of travel to the forest, parasitological confirmation using RDT should be offered prior to administration of anti-malarials.

After receiving confirmation of malaria by RDT, forest workers should have access to first line artemisinin combination therapy and primaquine. To accommodate this, health authorities should develop a clear mechanism for engaging with all registered private clinics that mostly visited by the ill health workers after office hours. These clinics must refer to the nearest primary health centre and receive appropriate malaria treatment. Lastly, importance of completing the full course of malaria treatment should be emphasized.

## Limitations

Participating workers provided estimated distances and locations of their forest activities. While maps were used to summarize this information, the accuracy of the locations provided is unknown. Interviewers and KIs were uncomfortable discussing illegal activities, which may have led to reduce emphasis on such themes and their potential impact on surveillance. Establishing rapport and practicing appropriate probing techniques for sensitive questions should be emphasized in future studies. This study examined forest workers in Aceh province, and therefore the findings may not be generalizable to other part of Indonesia. Nonetheless, the information gained from this formative study will useful for adapting RACD activities to mobile forest-working populations and in understanding the mobility patterns and shared risk factors that may link malaria transmission among the three areas of Aceh Besar and Aceh Jaya.

## Conclusions

All forest-working groups identified, such as farmers, cattle ranchers, loggers, miners, rattan gatherers and forest rangers are potentially exposed to malaria by working or sleeping overnight without protection and may be missed by passive surveillance. Some informants believed that male workers who work and stay in the forest are more vulnerable to contract with malaria compared to the villagers. Venue-based strategies appear to be feasible for miners, whilst peer-referral recruitment appears acceptable for farmers. Depending on their activities in the forest, a combination of venue-based and peer-referral recruitment may be suitable for loggers. These strategies should be rolled out to assess malaria infection risk and related behaviours among high-risk forest groups.

## Supplementary information

**Additional file 1.** Focus Group Discussion and In-Depth Interview Guideline.

## Data Availability

Not applicable.

## Abbreviations

API: Annual Parasite Incidence
ACD: Active Case Detection
FG: Focus Group
IDI: In-Depth Interview
IRS: Indoor Residual Spray
KI: Key Informant
LLIH: Long-Lasting Insecticidal Hammock
LLIN: Long-Lasting Insecticide-treated Net
NGO: Non-Governmental Organization
NIHRD: National Institute of Health Research and Development
PHC: Primary Health Centre
PR: Peer Referral
RACD: Re-Active Case Detection
RDT: Rapid Diagnostic Test
VB: Venue-Based
