# Supplementary material for: Defining malaria risks among forest workers in Aceh, Indonesia: a formative assessment
Source: Malar J. 2020 Nov 30;19:441. doi: 10.1186/s12936-020-03511-2 (PMC7708119; doi:10.1186/s12936-020-03511-2)
Supplement: Supplementary file 1 — Additional file 1. Focus Group Discussion and In-Depth Interview Guideline. [file 12936_2020_3511_MOESM1_ESM.docx]

**Supplementary files. Focus Group and In-Depth Interview Guideline**

**Focus Group Guide for Index Cases and Co-Workers**

1. **Malaria knowledge**
   1. What do you think are the most important symptoms of malaria?
   2. Who do you think is at highest risk of malaria in the community?
   3. Where would you say the majority of individuals infected with malaria come from?
2. **Forest activities**
   1. What are all of the reasons that people travel to forest or nearby the forest?
   2. Participatory Action Activity #1: Develop chart of forest and forest-fringe activities. Categories: Agricultural, mining, logging, gathering wood, foraging, and hunting. Discuss for each type of occupational/forest activity: location, resources involved, times of year, frequency, distance, length of travel time, travel alone or in group, length of stay, number of people in the group, local versus migrant workers, connection between workers, work organization.
3. **Mapping forest locations**
   1. Participatory Action Activity #2: Mapping of forest activities.
   2. Fill out the location listing for each spot identified
   3. How often do you see/hear macaques near the different sites? [Show picture]
4. **Health seeking**
   1. What do people do when they have fever?
   2. What kinds of places do they seek treatment?
   3. What are the reasons that people might not seek treatment?
   4. Are people working in illegal activities less likely to seek care at a primary health centre?
   5. Where would they seek care and why?
5. **Follow-up strategies**
   1. If we were interested in speaking with people who worked in the forest with someone who had been diagnosed with malaria, what do you think would be the best way to find them?
6. **Location-based screening**
   1. Would it be possible to interview and test people at their work sites?
   2. Are there other locations where forest labourers tend to gather or meet?
   3. Do you think that it is possible to map all such locations*?*
7. **Peer-referral screening**
   1. Do most workers tend to know one another? In what contexts do they interact? Are there any groups that do not interact with each other?
   2. Do you think it would be feasible to contact one another by mobile phone or through the village midwives/employers?
   3. If we were to contact people this way and ask them to come for an interview or malaria screening, what would be the best places to locate interviews/screening sites?
   4. Are there certain interview/screening hours that would be best? What kind of person should conduct interview/screening? Which languages would they need to speak?
8. **Participating in a future malaria study**
   1. Would you be interested in participating in a study with malaria testing?
   2. We are interested in how often people go into the forest, how long they stay and far they travel so we can learn about their malaria risk. Would you be comfortable with the idea of carrying a GPS tracker in your pocket, all the time, for up to a month?
   3. Do you think that any kinds of forest workers may be hesitant to present to a screening centre in a mosque or health centre for any reason? For example, unfamiliarity, privacy concerns, etc.
   4. What is the cost of travelling from the forest to the health centre? If you were asked to come for an interview and screening, would you come if you received 50,000 IDR compensate you for your time?

**Focus Group Guide for Community Health Workers**

1. **Malaria knowledge**
   1. What do you think are the most important symptoms of malaria?
   2. Who do you think is at highest risk of malaria in the community?
   3. Where would you say the majority of individuals infected with malaria come from?
2. **Access to health services and treatment-seeking**
   1. Where are all of the places that people seek treatment when they have fever?
   2. Participatory Activity #1: Complete chart of providers
   3. What are the reasons that people might not seek treatment when they have a fever? Why would they choose to seek treatment in these different places?
   4. Are there certain providers that specific groups of people are more likely to visit?
   5. What about those you identified as high risk of malaria (recap) that was mentioned before?
   6. Do you serve any migrants? If so, where do they come from?
3. **Malaria prevention**
   1. What malaria prevention measures do people use in the community?
   2. What about those who you previously identified as having high risk of malaria?
   3. What are some of the challenges people face in accessing bed nets and IRS?
   4. What do you think are some ways to improve access to malaria prevention and treatment in the community? What about in these high-risk populations?
4. **Follow-up strategies**
   1. If we were interested in finding people who worked in the forest with someone who had been diagnosed with malaria, what do you think would be the best way to find them?
   2. Two different ways of finding these people have been suggested. One is to go to a work site where we think the case was infected and screen people who work there. The other way is to get contact information (like telephone numbers or village name) of people who worked in the forest with the malaria case. What do you think would be opportunities and challenges using these two approaches?
5. **Participating in a future malaria study**
   1. Do you think that people in the community would be interested in participating in a study with malaria testing?

**Focus Group Guide for Community Members**

1. **Malaria knowledge**
   1. What are the main symptoms of malaria?
   2. Are there specific groups of people in the community that you think are at high risk of malaria?
   3. Where would you say the majority of individuals are infected with malaria?
   4. How do you distinguish between forest and forest-fringe areas?
2. **Occupational and forest related exposures**
   1. What kind of activities do most people who live around here do to generate income?
   2. Are there any other kinds of activities that people do outdoors between dusk to dawn?
   3. Do people around here travel outside of the district to find work?
   4. What are all of the reasons that people travel to forest or forest-fringe areas in the district? What about outside of the district?
3. **Follow-up strategies**
   1. If we were interested in finding people who worked in the forest with someone who had been diagnosed with malaria, what do you think would be the best way to find them?
   2. Two different ways of finding these people have been suggested. One is to go to a work site where we think the case was infected and screen people who work there. The other way is to get contact information (like telephone numbers or village name) of people who worked in the forest with the malaria case. What do you think would be opportunities and challenges using these two approaches?
4. **Prevention and treatment-seeking behaviour**
   1. Where do people seek treatment when they have symptoms of malaria and why?
   2. What are the main challenges for people to access malaria treatment when they are ill?
   3. What malaria prevention measures do people use in the community?
   4. What are some of the challenges people face in accessing bed nets and IRS?
   5. What do you think are some ways to improve access to malaria prevention and treatment in the community?
5. **Participating in a future malaria study**
   1. Do you think that people in the community would be interested in participating in a study with malaria testing?
   2. We are interested in how often people go into the forest, how long they stay and far they travel so we can learn about their malaria risk. Do you think that people would be comfortable with the idea of carrying a GPS tracker in their pocket, all the time, for up to a month?

**In-depth Interview Guide for Malaria Patient**

1. **Malaria knowledge**
   1. What are the main symptoms of malaria?
   2. Who do you think is at highest risk of malaria in the community
   3. Where would you say the majority of individuals infected with malaria come from
2. **Occupational and forest related exposures**
   1. What kind of activities do you do to generate income?
   2. Are there any other kinds of activities that you do outdoors between dusk to dawn?
   3. Do you travel to areas outside of the district to find work?
   4. What are all of the reasons that you travel to forest or forest-fringe areas in the district? What about outside of the district?
   5. Participatory Action Activity #1: Develop chart of forest/fringe activities & crops, seasonality and frequency. Categories: Agricultural, logging, gathering wood, foraging, and hunting. Discuss for each type of occupational/forest activity: location, mode of transportation, length of stay, forest dwelling, times of year, frequency, distance, length of travel time, travel alone or in group, length of stay, number of people in the group, local versus migrant workers, connection between workers, work organization and willingness to be interviewed.
   6. Participatory Action Activity #2: Mapping of forest activities – location and type of activities.
   7. How often do you see macaques near the different sites? [Show picture]
3. **Follow-up strategies**
   1. If we were interested in finding people who worked in the forest with someone who had been diagnosed with malaria, what do you think would be the best way to find them?
4. **Location-based screening**
   1. Are there locations where forest laborers tend to gather or meet? What are all of the kinds of locations?
   2. Do you think it is feasible to approach people for interview and blood test at work sites and other gathering locations?
5. **Peer-referral screening**
   1. Are there different groups of people who work in the forest: for example, do some people interact or know each other and others not?
   2. Do you think it would be feasible to contact one another by mobile phone or through the village midwives/employers?
   3. If we were to contact people this way and ask them to come for an interview or malaria screening, what would be the best places to locate interviews/screening sites?
   4. Are there certain interview/screening hours that would be best? What kind of person should conduct interview/screening? Which languages would they need to speak?
6. **Prevention and treatment-seeking behaviour**
   1. Where do you seek treatment when you have symptoms of malaria and why?
   2. What are the main challenges you face in accessing malaria treatment when you are ill?
   3. What malaria prevention measures do you use at home?
   4. What are some of the challenges you face in accessing bed nets and IRS?
   5. What do you think are some ways to improve access to malaria prevention and treatment?
7. **Participating in a future malaria study**
   1. Would you be interested in participating in a study with malaria testing?
   2. We are interested in how often people go into the forest, how long they stay and far they travel so we can learn about their malaria risk. Would you be comfortable with the idea of carrying a GPS tracker in your pocket, all the time, for up to a month?
   3. Do you think that any kinds of forest workers may be hesitant to present to a screening centre in a mosque or health centre for any reason?
   4. What daily allowance do you receive for a day’s work?
   5. What is the cost of travelling from the forest to the health centre?

**In-depth Interview Guide for Forest Employers**

1. **Malaria knowledge**
   1. What are the main symptoms of malaria?
   2. Who do you think is at highest risk of malaria in the community?
   3. Where would you say the majority of individuals infected with malaria come from?
2. **Working conditions**
   1. Please tell us about the operations that you run in this area.
   2. Can you describe a typical day for a forest worker who you employ for this kind of activity?
   3. Can you describe a typical site(s) where workers would be based for this kind of work?
   4. Are there conditions of work that might lead to malaria? Please elaborate.
   5. What type of housing do workers use when they are staying in the forest?
   6. If a worker has a fever, where do they go to seek treatment? What kind of transportation would they use to get there?
3. **Recruitment**
   1. How do you usually recruit workers?
   2. Are there specific locations where people go to be recruited for these jobs? If so, where are these places?
   3. Where do the workers originate? (ie. Local residents or from other sub districts/districts)
   4. How many hours on average do workers travel to work at these sites?
   5. What kind of transportation do workers use to come to these sites?
   6. How many people typically travel together to the work sites? (number)
4. **Location and timing**
   1. What hours of the day are laborers working?
   2. How far from the main site do workers travel during operations? Does this area differ from the site where they sleep in terms of land cover, remoteness, etc?
   3. What is the length of time/duration one group of people stays at site?
   4. When and how often do they tend to return home?
5. **Follow-up strategies**
   1. Do you think that you, or other people employing laborers for this kind of work, would be supportive of a malaria study amongst your workers?
   2. If we were to contact people this way and ask them to come for an interview or malaria screening, what would be the best places to locate interviews/screening sites?
   3. Are there certain interview/screening hours that would be best? What kind of person should conduct interview/screening? Which languages would they need to speak?
   4. Outside of the workplace, are there locations where forest laborers tend to gather or meet?
   5. Do you think that workers would be interested in participating in a study with malaria testing?
   6. What compensation, if any, would be needed to encourage participation?
6. **Peer-referral screening**
   1. Are there different groups of people who work in the forest: for example, do some people interact or know each other and others not?
   2. Do you think it would be feasible to contact one another by mobile phone or through the village midwives/employers?

**In-depth Interview Guide for Community Leaders or Non-Government Office (NGO)**

1. **Background**
   1. Can you please describe your role in this community/organization?
   2. NGO only: How do you work with communities and what services do you provide?
   3. What kind of activities do most people who live around here do to generate income?
   4. Do people around here travel to other areas to find work?
2. **Malaria situation in Aceh**
   1. Please tell me about the situation with malaria in Aceh.
   2. Are there specific groups of people in the community that you think are at high risk of malaria?
   3. Where would you say the majority of individuals are infected with malaria?
3. **Occupational and forest-related exposures**
   1. What kind of activities do most people who live around here do to generate income?
   2. Do people around here travel outside of the district to find work?
   3. What are all of the reasons that people travel to forest or forest-fringe areas in the district? What about outside of the district?
4. **Other forest activities**
   1. Are there forest activities that people might be unwilling to report in a malaria study?
   2. Are people working in these illegal activities less likely to seek care at a puskesma? Where would they seek care and why?
5. **Follow-up strategies**
   1. If we were interested in finding people who worked in the forest with someone who had been diagnosed with malaria, what do you think would be the best way to find them?
6. **Location-based screening**
   1. Are there locations where forest laborers tend to gather or meet? What are all of the kinds of locations?
   2. Do you think it is feasible to approach people for interview and blood test at work sites and other gathering locations?
7. **Peer-referral screening**
   1. Do you think it would be feasible to contact people who work with a case by mobile phone or through the village midwives/employers?
   2. If we were to contact people this way and ask them to come for an interview or malaria screening at a site near them, what would be the best places to locate interviews/screening?
   3. Are there certain interview/screening hours that would be best? What kind of person should conduct interview/screening? Which languages would they need to speak?
   4. Do you think that any kinds of forest workers may be hesitant to present to a screening centre in a mosque or health centre for any reason?
8. **Prevention and treatment-seeking behaviour**
   1. Where do people seek treatment when they have symptoms of malaria and why?
   2. What are the main challenges for people to access malaria treatment when they are ill?
   3. What malaria prevention measures do people use in the community?
   4. What are some of the challenges people face in accessing bed nets and IRS?
   5. What do you think are some ways to improve access to malaria prevention and treatment in the community?
9. **Participating in a future malaria study**
   1. Do you think that people in the community would be interested in participating in a study with malaria testing?
   2. Do you think that any kinds of forest workers may be hesitant to present to a screening centre in a mosque or health centre for any reason?
   3. We are interested in how often people go into the forest, how long they stay and far they travel so we can learn about their malaria risk. Do you think that people would be comfortable with the idea of carrying a GPS tracker in their pocket, all the time, for up to a month?
   4. What daily allowance do people working in the forest receive for a day’s work? What is the cost of travelling from the forest to the health centre?

**In-depth Interview Guide for Health Facility Staff**

1. **Background**
   1. Can you please describe your role?
   2. What kind of activities do most people who live around here do to generate income?
   3. Do people around here travel to other areas to find work?
2. **Malaria in Aceh**
   1. Please tell me about the situation with malaria in Aceh
   2. Are there specific groups of people in the community that you think are at high risk of malaria?
   3. Where would you say the majority of individuals are infected with malaria?
3. **Occupational and forest-related exposures**
   1. What kind of activities do most people who live around here do to generate income?
   2. Do people around here travel outside of the district to find work?
   3. What are all of the reasons that people travel to forest or forest-fringe areas in the district? What about outside of the district?
4. **Other forest activities**
   1. Are there forest activities that people might be unwilling to report in a malaria study?
   2. Are people working in these illegal activities less likely to seek care at a puskesma? Where would they seek care and why?
5. **Access to health services and treatment-seeking**
   1. Where are all of the places that people seek treatment when they have fever?
   2. Participatory Activity #1: Complete chart of providers – For each type of place, discuss: type of facilities, availability of malaria treatment, sequence of events in the health provider.
   3. What are the reasons that people might not seek treatment when they have a fever? Why would they choose to seek treatment in these different places?
   4. Are there certain providers that specific groups of people (such as miners or farmers, or loggers) are more likely to visit? What about those you identified as high risk of malaria (recap) that you mentioned before?
   5. Do you serve any migrants (people who do not live here permanently, but are travelling through for short periods of time)? If so, where do they come from?
6. **Treatment strategies**
   1. Do you currently have primaquine in stock at this health facility?
   2. What is the most frequent dose that you give to malaria patients?
   3. Do you always prescribe primaquine for all malaria patients? Do you always prescribe primaquine to cases of *P. vivax*? If no, why?
   4. Why do you think the reasons why primaquine should be given to malaria patients?
   5. Do you think malaria patients will completely finish their course of primaquine for *P. vivax*? How could you possibly know whether your patients take or not take the primaquine completely?
   6. What are your concerns around the use of primaquine?
   7. Have you ever heard about G6PD testing? Could you please describe it?
7. **Malaria prevention**
   1. What malaria prevention measures do people use in the community? What about those who you previously identified as having high risk of malaria?
   2. What are some of the challenges people face in accessing bed nets and IRS?
   3. What do you think are some ways to improve access to malaria prevention and treatment in the community? What about in these high-risk populations?
8. **Follow-up strategies**
   1. If we were interested in finding people who worked in the forest with someone who had been diagnosed with malaria, what do you think would be the best way to find them?
   2. Two different ways of finding these people have been suggested. One is to go to a work site where we think the case was infected and screen people who work there. The other way is to get contact information (like telephone numbers or village name) of people who worked in the forest with the malaria case. What do you think would be opportunities and challenges using these two approaches?
9. **Participating in a future malaria study**
   1. Do you think that people in the community would be interested in participating in a study with malaria testing?
   2. What incentives, if any, would be needed to encourage participation? How can manipulation be avoided?
